# Cultural practices, oral health service utilisation and oral health policy and guidelines development in Africa: insights from the yorùbá ethnic group

**DOI:** 10.3389/froh.2025.1539827

**Published:** 2025-02-19

**Authors:** Moréniké Oluwátóyìn Foláyan, Oluwabunmi Tope Bernard, Olusegun Stephen Titus, Omolola Alade, Taofeek Kolawole Aliyu, Ahmed Bhayat, Nicaise Ndembi, Gbenga Fasiku, Maha El Tantawi

**Affiliations:** ^1^ The Africa Oral Health Network (AFRONE), Alexandria University, Alexandria, Egypt; ^2^ Department of Child Dental Health, Obafemi Awolowo University, Ile Ife, Nigeria; ^3^ Department of Languages and Cultures, Ghent University, Ghent, Belgium; ^4^ Department of Music, Obafemi Awolowo University, Ile Ife, Nigeria; ^5^ Department of American Studies, Konstanz University, Konstanz, Germany; ^6^ Department of Preventive and Community Dentistry, Faculty of Dentistry, College of Health Sciences, Obafemi Awolowo University, Ile Ife, Nigeria; ^7^ Department of Sociology and Anthropology, Obafemi Awolowo University, Ile-Ife, Nigeria; ^8^ Department of Community Dentistry, University of Pretoria, Pretoria, South Africa; ^9^ Division of Epidemiology and Prevention, Institute of Human Virology, University of Maryland School of Medicine, Baltimore, MD, United States; ^10^ Department of Philosophy/Institute of Cultural Studies, Obafemi Awolowo University, Ile Ife, Nigeria; ^11^ Department of Paediatric Dentistry and Dental Public Health, Alexandria University, Alexandria, Egypt

**Keywords:** indigenous, decolonisation, cultural sensitivity, oral hygiene, traditional practice, Africa

## Abstract

Cultural beliefs and practices influence oral health behaviors in Africa, where traditions around health are deeply woven into daily life and community values. From the symbolism embedded in African art, belief in efficacy of herbs and natural elements, to the communal customs around oral hygiene, culture serves as a lens through which health beliefs and practices related to oral health are cultivated, understood and acted upon. This manuscript discusses rituals and embodied culture practices surrounding oral health among yorùbá, and links this discussion to the implications for oral health policies and interventions in Africa. Yorùbá is a prominent ethnic group in West Africa with oral traditional practices which reflect the community's emphasis on natural remedies, often reducing reliance on professional dental care. Through an analysis of oral health “rituals” embedded in yorùbá songs, proverbs, and Ifá divination poetry, this manuscript argues that cultural expressions reinforce the importance of oral health as a manifestation of spiritual balance and personal oral hygiene. Despite this cultural reverence, the manuscript finds that dental service utilization remains low among the yorùbá, with spiritual and traditional interpretations of oral health concerns often delaying professional intervention. It further suggests that integrating culturally resonant strategies into oral health policies could bridge gaps in service uptake. By analyzing these dynamics, the study offers a culturally informed perspective on oral health research and proposes policy frameworks that integrate indigenous and modern health approaches.

## Introduction

Oral health disparities are a significant public health issue across Africa, with oral diseases affecting 1 in 3 persons in the World Health Organisation's Africa Region (1). The risk for poor oral health is exacerbated by the poor access to preventive and therapeutic dental care in the region (2). The limited availability of resources, combined with systemic healthcare challenges, has further left many African communities underserved by oral health care services (3). Yet, Africa's oral health landscape is uniquely shaped by rich and diverse cultural practices, beliefs, and values that can have a profound impact on health (4), influencing how individuals approach oral hygiene, dietary habits, and the utilization of dental services (5).

Culture is often understood as the shared patterns of beliefs and behaviors within specific groups, offering frameworks that shape social norms, relationships, and rules of conduct (6). Modern discourse tends to emphasize its observable components—tangible elements shared and transmitted within societies (7). However, because these aspects are mutable and no cultural trait is exclusive to any group, culture encompasses far more than its visible elements. In today's global cultural “market,” where traits are freely adopted and adapted, the essence of culture extends beyond what is observable (8).

A key dimension of culture lies in the structured ways individuals interpret their world, shaped by both conscious and unconscious beliefs, expectations, and practices. The essence of culture is not found in artifacts or tangible elements but in the values, symbols, and perspectives that define a group's identity. As noted, “the essence of a culture is not its artifacts, tools, or other tangible elements but in how group members interpret, use, and perceive them. It is the values, symbols, interpretations, and perspectives that set one people apart from another in modernized societies; not the material objects or other tangible aspects of societies (8).”

Hence, it is essential that the intrinsic elements of culture reveals its deeper essence, which imbues visible expressions with meaning. These core elements enable a culturally conscious individual to recognize, embrace, and defend their culture's visible expressions. Integral to this inner essence are the beliefs, shared mental states, and values shaped by a community's traditions and passed down through generations. They act as the nonhereditary memory of a community, expressed through systems of constraints and prescriptions (9). So, an understanding of culture as the patterned way individuals interpret their world, relying on both conscious and unconscious assumptions, expectations, and practices, forms a theoretical framework for this paper. These cultural frameworks influence how groups perceive illness and approach healthcare, sometimes facilitating access to services but occasionally creating barriers (10, 11).

Arthur Kleinman's “explanatory model” highlights that each culture has its own ways of understanding health and illness, encompassing biological, social, spiritual, and moral dimensions (12). These beliefs are “embodied” in physical practices and expressions, making oral health a cultural expression. Embodiment of oral health can be understood as an act of “doing oral health,” as it involves learning, adapting, and performing practices related to maintaining oral hygiene and care. Embodiment is not a static concept; rather, it is “a life process that requires the learning of body techniques such as brushing, flossing, chewing, and speaking (13).” It is through the integration of these oral health practices that one constructs and sustains the sense of bodily care and health within everyday life. In many African societies, the details of the mouth such as the appearance of teeth, influences social perceptions, like associating white, well-maintained teeth with beauty, health, and status.

Despite the significance of cultural beliefs for oral health, and the need for cultural sensitivity to improve access and uptake of oral health services (14), oral health policies in Africa have often neglected to meaningfully incorporate this cultural consciousness, leading to a gap in culturally resonant health strategies. Culturally competent health care providers are better equipped to meet the needs of community members, who are often underserved, by respecting their unique beliefs, values, language, practices, and health behaviors (15).

The gap may be due, in part, to a longstanding influence of colonial healthcare models, which historically marginalized traditional and indigenous African healthcare systems and replaced them with Western-centric perspectives (16). The transition diminished the use of local remedies and community-based health practices, valuable knowledge, cultural heritage, and self-sufficiency in oral health care, resulting in poor integration of indigenous and local knowledge, fostering dependence on biomedical models that prioritize symptom treatment over a holistic indigenous approach that considers physical, mental, and spiritual well-being (17).

The legacy of these shifts often resulting from colonisation and reinforced by settler colonisation practices, have shaped current public health policies and healthcare education, often creating a bias toward Western approaches at the expense of cultural relevance (18, 19). In many cases, oral health professionals and policymakers lack training in culturally sensitive care and may have limited understanding of the sociocultural contexts within which communities interpret and manage their health, including oral health (20).

Fifty-five countries are members of the African Union (21) with many more ethnic groups and their unique cultures and ethnicities. It is unrealistic to talk about the “African” culture or to generalise the intricate tapestry of cultural beliefs of one group to all Africa. In this respect, we focus on one ethnic group in West Africa, the yorùbá people, to demonstrate the complexity of the interaction between cultural beliefs, traditional practices and oral health, and the possible impact of their unique worldview on oral health. This paper discusses rituals and embodied culture practices surrounding oral health, and links this discussion to the implications for oral health policies and interventions in Africa.

## Yorùbá and traditional value for oral health

The yorùbá are one of West Africa's prominent ethnic groups, primarily residing in southwest Nigeria and parts of neighboring countries like Benin and Togo (22). They are renowned for their rich cultural heritage, which encompasses a complex social structure, language, religion, literature, and arts. Yorùbá culture is deeply rooted in spirituality and cosmology, with the traditions passed down for centuries through oral histories, art, and the sacred *Ifá* corpus—a system of divination that provides philosophical insights on various aspects of life, including health and wellness (23). The yorùbá, like all cultures, find metaphysical explanations for anything they cannot rationally explain, but are also more likely than other ethnic groups in Nigeria to link metaphysical causes to oral diseases (24).

In yorùbá society, the mouth holds a profound symbolic importance, reflecting not only a physical but also a spiritual dimension. It is seen as the “gateway” to expressing one's inner character, used for speech, prayers, and blessings (25). The yorùbá worldview on health centers around a holistic approach, with the concept of personhood comprising *ara* (the body), *ẹ̀mí* (the life force or soul), and *orí* (the inner head or spiritual essence). Each element is thought to contribute to an individual's health, fate, and balance in life. As a result, the mouth's care is seen as integral to nurturing the *ẹ̀mí* and harmonizing the *orí* with the physical self (26). This reverence is reflected in proverbs and maxims, which emphasize the mouth's role in maintaining personal integrity, reputation, and aligning one's inner and outer worlds (25).

Furthermore, an *ẹsẹ-Ifá* (Ifá divination poem), the *Òdí Méjì,* foregrounds the importance of the appearance of the teeth in determining the state of human health. *Òdí Méjì* emphasizes that dazzling white teeth is synonymous with good health which is manifested in a healthy body. Since the yorùbá worldview on health centers on maintaining balance among *ara*, *ẹ̀mí*, and *orí* (particularly because it also houses the teeth), *Òdí Méjì* emphasizes that healthy teeth are a physical manifestation of spiritual balance of the three elements, and that only dazzling and sparking appearance fits the teeth (*funfun niyì eyín*) (27).

This belief in the spiritual and physical balance evident in the healthy teeth, encourages yorùbá people to engage in regular oral health rituals. The need for these rituals is reinforced through the yorùbá verbal arts for indigenous health management. Verbal arts keep people well-informed of the beliefs, convictions, and the aspiration of the yorùbá traditional setting (28). Among the verbal arts, folksongs have been identified as a medium of continuity in preserving the traditional values and norms in African society and culture (29). Traditional yorùbá songs, proverbs, and chants carry messages that celebrate good health practices, including oral hygiene, and link these practices to overall balance and harmony in life. Through their rhythm, repetition, and cultural significance, these songs remind individuals of the importance of maintaining healthy mouth, teeth, and tongue—not only for physical health but also for spiritual and mental wellness.

In many yorùbá songs, bright, clean teeth and a well-cared-for mouth are associated with good fortune, spiritual clarity, and respectability. They often echo the belief that physical appearance, such as dazzling white teeth, reflects inner spiritual balance and strength. For instance, lyrics might emphasize that “*eyín funfun*” (white teeth) are a source of “*iyì*” (honor or dignity), suggesting that oral health is both a visible sign of personal integrity and a reflection of one's spiritual and mental state.

**Figure 1:**
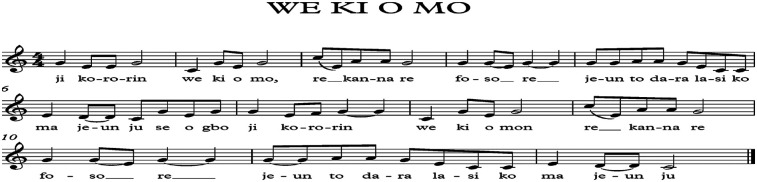
Lyrics of the popular yorùbá song titled: “we Ki O Mo”.

One of such songs known and popularly sung among the yorùbá people is titled “wẹ*`* kó o mọ*´*.” Jí ko rorín, wẹ*`* kí o mọ*´*, ré èékánná rẹ, jẹun tí ó dára lásìkò má jẹun jù (Wake up and clean your teeth, have a bath to stay clean, cut your fingernails, eat good food at the right time, but do not overeat). The lyrics is shown in Figure 1.

The song reflects personal integrity by linking physical hygiene, discipline, and well-being to yorùbá values of dignity and self-care. It emphasizes daily practices like bathing, brushing, and nail-cutting as symbols of inner discipline and respect for oneself and others. In the yorùbá culture, outward cleanliness represents inner values, aligning physical care with moral and social responsibility. The call to “eat good food and don't be a glutton” highlights moderation as a core yorùbá virtue. Balanced eating reflects self-control and thoughtful living, resonating with the philosophy of *ìwà pẹ̀lẹ́* (gentle and disciplined character). Overindulgence, by contrast, signifies a lack of restraint.

In yorùbá cosmology, the body (*ara*) houses the spirit (*ẹ̀mí*) and is guided by the spiritual essence (*orí*). Caring for the body fosters spiritual and mental clarity, maintaining harmony and balance, aligning with cultural expectations of purity and holistic health. Songs like this educate by embedding values of self-care, moderation, and responsibility, transmitting yorùbá cultural norms that connect health, morality, and spirituality. These kinds of songs are often used in communal gatherings, serve as tools for educating children and adults alike, and encourages listeners to reflect on how their daily habits align with their personal values and communal expectations. It preserves and transmits yorùbá cultural norms, emphasizing the interconnectedness of health, morality, and spirituality, thereby creating a harmonious state that honors the yorùbá understanding of total wellness.

Thus, the yorùbá worldview of the mouth is that of both a physical and spiritual symbol, connecting oral health to broader concepts of personhood. Oral health is, therefore, a holistic embodiment of spiritual balance and inner harmony. The mouth becomes a “gateway” reflecting character and spiritual health, embodying cultural values beyond physical care. The culture reinforces the mouth's role in maintaining personal integrity and aligns physical health with moral and spiritual ideals. This may clarify why the yorùbá people place considerable value on oral health practices as the mouth's care is not simply a matter of hygiene or aesthetics but an essential practice for maintaining physical, spiritual, and social balance.

## Cultural inundated reasons for low dental service utilisation in Southwest Nigeria

Despite the cultural reverence for the mouth and the need to keep it healthy, dental service utilization remains low in southwest Nigeria, where the yorùbá population is concentrated. A rapid review of the literature was applied for publications in English addressing dental service utilization in Nigeria was conducted. A search was conducted in Pubmed utilizing keywords and Medical Subject Headings (MeSH) terms. The first set of terms include “dental service”, “oral health care” separated by Boolean operator OR. The second set included the term “access”, “utilization” separated by Boolean operator “OR” and the third set included the term “Nigeria.” Primary studies focusing on dental service utilisation in any of the six states in southwest Nigeria (Lagos, Ondo, Ekiti, Ogun, Osun and Oyo States) were eligible for inclusion in the review. The references of the included studies were further reviewed for additional publications. The primary outcome assessed was the percentage of study participants that utilised dental services. After abstract screening and full text reviews, 14 articles were identified (30–43). The data on author and year of publication, target and age of the target population for the study, and the sample size were extracted and summarized shown in Table 1.

**Table 1:** Publications on dental service utilization in South West Nigeria.

Name of first author	Year of publication	Title of manuscript	Target population	Age of target population	Sample	Percentage using dental services in the study
Adegbembo (30)	1994	Household Utilization of Dental Services in Ibadan, Nigeria	Children and adolescents	6–18 years	1,393	8.8%
Bamise (31)	2008	Dental Care Utilization and Satisfaction of Residential University Student	University students	16–48 years	605	7.8%
Oredugba (32)	2008	Use of oral health care services and oral findings in children with special needs in Lagos, Nigeria	Children with special needs	5–19 years	243	<5%
Akaji (33)	2008	Utilization of dental services among secondary school students in Lagos, Nigeria	Secondary school students	10–19 years	502	14.9%
Ajayi (34)	2012	Barriers to Oral Health Care Utilization in Ibadan, South West Nigeria	Patients	16–78 years	400	27.3%
Ola (35)	2012	Family Structure, Socioeconomic Position and Utilization of Oral Health Services Among Nigerian Senior Secondary School Pupils	Senior secondary school pupils	Mean age: 15.8 (1.9) years	1,043	22.5%
Opeodu (36)	2012	Dental Health Service Utilisation by Resident Doctors/Medical Officers in The University College Hospital, Ibadan, Oyo State, Nigeria	Medical doctors		190	65.3%
Folayan (37)	2013	Factors limiting dental service utilization by pupils in Ile-Ife, Nigeria. Nigeria Journal of Health Sciences.	Secondary school students	9–12 years	139	15.8%
Osuh (38)	2014	Dental Services and Attitudes Towards Its Regular Utilization Among Civil Servants in Ibadan, Nigeria	Civil servants	25–61 years	400	39%
Esan (39)	2015	Effect of a school-based oral health education programme on use of recommended oral self-care for reducing the risk of caries by children in Nigeria.	School children	Information not available	241	11.2%
Adedigba (40)	2016	Pattern of Utilisation of Dental Health Care Among HIV-Positive Adult Nigerians	People living with HIV	18–70 years	239	7%
Olatosi (41)	2020	Maternal Knowledge, Dental Service Utilization and Self-Reported Oral Hygiene Practices in Relation to Oral Health of Preschool Children in Lagos, Nigeria	Mothers of children under-five	31–47 years	334	21.9%
Adeniyi (42)	2020	Predisposing, Enabling and Need Factors Influencing Dental Service Utilization Among A Sample of Adult Nigerians	General population	18–80 years	400	39.2%
Folayan (43)	2020	Association Between Maternal Socioeconomic Factors, Decision-Making Status, And Dental Utilization by Children, With Early Childhood Caries in Sub-Urban Nigeria	Children in the general population	1–5-years	1,549	5.9%

The included studies encompass diverse demographic groups, spanning different ages, social structures, and health conditions, with sample sizes ranging from 139 to 1,549 participants. These studies offer insights into dental service utilization across varied populations. Several studies highlighted low utilization rates among younger populations: only 8.8% of children and adolescents aged 6–18 years in Ibadan utilized dental services (30), and the rate was even lower at 5.9% among children aged 1–5 years in Ile-Ife (43). This underscores significant barriers to access and utilization in paediatric populations, particularly for vulnerable groups like children with special needs, who had utilization rates of less than 5% (32).

Utilization among secondary school students ranged from 14.9% in Lagos (33) to 15.8% in Ile-Ife (37). University students reported even lower rates, with only 7.8% utilizing dental services (31). Special populations also exhibited low rates of utilization, including people living with HIV (7%) (40) and mothers of young children (21.9%) (41). In contrast, studies focusing on adults, particularly professionals, reported higher utilization rates. For example, 65.3% of resident doctors (36), 39% of civil servants (38), and 39.2% of the general adult population (42) accessed dental services.

Overall, the findings reveal that dental service utilization in Southwest Nigeria remains generally low, with significant disparities among population groups. Children, adolescents, and economically disadvantaged groups are particularly underserved, while professionals and individuals with greater financial and educational resources are more likely to access dental care. Identified barriers include socioeconomic status and family structure, both of which significantly influence utilization (34, 35).

Intervention efforts showed limited impact, as demonstrated by the modest 11.2% utilization rate following a school-based oral health education program (39). This suggests that while such interventions may be beneficial, broader structural changes and enhanced support systems are necessary to achieve meaningful improvements in dental service utilization. A deeper understanding of how culturally tailored interventions can provide structural changes and enhanced support systems may help address this persistent problem that had been observed since 1994 and still persists till date.

### Cultural emphasis on traditional remedies

In yorùbá culture, many individuals may prioritize natural and home-based remedies over modern medical care. The use of chewing sticks, such as *pákò* and o*rín*, derived from local plants with antimicrobial properties is common (44). These sticks are believed to clean the mouth, promote periodontal health, and offer spiritual protection (45, 46). The perception that chewing sticks suffice for oral hygiene may reduce actual seeking of professional dental care, particularly for preventive purposes. This reliance on traditional practices to support health is often reinforced by generational knowledge passed down through families (47). The approval by the community as an acceptable norm makes the narrative a strong influence on health behaviors (48).

### Spiritual and social perceptions of oral health

The mouth is seen as a reflection of spiritual balance in the yorùbá culture. As such, oral diseases might be seen through a spiritual lens rather than being viewed as medical conditions that require professional intervention. For example, gum swelling or mouth sores could be attributed to spiritual imbalances or external spiritual forces, similar to what western scholars may interpret as the individual having an external locus of control (49, 50). This spiritual interpretation of oral health may lead individuals to seek traditional or spiritual remedies instead of visiting a dentist, thus fostering a delayed response to oral problems. Many yorùbá individuals may seek intervention only when pain or visible symptoms escalate, but may still initially resort to culturally rooted beliefs or practices that delay professional treatment (51).

In addition, many cultures prioritize health issues that cause significant pain or impair daily functioning. In this case, preventive dental care is often perceived as less essential than other medical needs, and people only seek dental care for emergencies because they consider routine preventive care to be unnecessary or secondary.

### Mistrust of western medicine and health care services

Historical influences, particularly the legacy of colonialism, have affected perceptions of Western medicine (52). Traditional healers, who are familiar with local customs and spiritual practices, are often trusted more than Western-trained professionals (53–55). Hospitals and clinics might feel foreign to those who identify more with indigenous methods as traditional medical practitioners often share the same language and worldview, with health and illness perceived in the same light (56). The perception that Western medicine lacks cultural sensitivity can discourage individuals from utilizing dental services, especially if dental visits are perceived as more intimidating than traditional care methods.

To address these concerns, the integration of orthodox and traditional health practices within public and private health facilities should be actively promoted in Africa through policy development. The World Health Organization had urged governments to include traditional medical practitioners in primary health care. This integration was officially endorsed in the 1978 Alma Ata Declaration and the Declaration of Astana 2018, which emphasized the role of traditional practitioners alongside other health workers in meeting community health needs, and the appropriate use of traditional medicine as one of the strategies to achieve universal health coverage respectively. These calls were made in view of the huge potential that integration holds for encouraging the continuity of healthcare (57) and enhancing primary healthcare delivery (58, 59). A number of countries in Africa have adopted national policies and regulations for integrated practices (60), including Nigeria, Sierra Leone and Ghana.

### Self-reliance and oral health care

Self-management of oral health in the yorùbá community is strongly aligned with cultural beliefs, emphasizing self-reliance, spirituality, and natural remedies. Chewing sticks, mentioned earlier, have medicinal and spiritual value (61), serving as natural tools for oral hygiene among those who prefer a plant-based, non-invasive care (62). Similarly, herbal rinses and saltwater gargles are seen as harmonizing with the body's natural state (63), reinforcing cultural values of balance and respect for one's spiritual essence, or *orí*. The yorùbá community's deep-seated value for self-reliance and resilience may mean that people try to manage discomfort or minor dental issues on their own, seeking professional care only as a last resort.

Studies on the low utilisation of dental services had highlighted the high level of self-care practices for oral health care, the pluralistic health system with the Western healthcare that are predominantly urban-centered competing with traditional and self-care approaches, and the alternative source of care provided by traditional healers which are not duly acknowledged (30). There are, however, no studies exploring the nexus of self-reliance, culture and dental service utilisation in general, and in African populations specifically.

However, while self-management practices resonate culturally, they may not fully address complex oral health needs, such as cavities or periodontal disease, that require modern interventions like fluoride and professional cleanings. This may explain why patients report late to modern healthcare facilities for oral care (64). When patients present to the healthcare facilities, this is probably not their first attempt at accessing care: they are likely to have come to these facilities after exhausting their cultural solutions to the problem. Western-trained healthcare providers often lack awareness of how the cultural perspective of self-management influences health behaviors, potentially misinterpreting delayed facility use as neglect or lack of awareness, rather than as a culturally informed choice.

### Stigma and social perceptions of illness

Oral health issues can carry a degree of social stigma within the yorùbá community, where the appearance and health of the mouth are tied to social and spiritual integrity. Conditions like dental caries or halitosis may be perceived as signs of poor personal management or spiritual imbalance, leading some individuals to conceal these issues rather than seek help (65). This stigma may be particularly intense for individuals with conditions perceived to carry a spiritual or social failing, leading to avoidance of dental services to prevent judgment or gossip.

## Implications for health policy and intervention

Oral healthcare among communities with deep rooted cultures may require culturally sensitive health policies that consider the cultural perspectives of oral health. As the study of the yorùbá culture may suggest, public health campaigns can benefit from involving local leaders, traditional healers, and respected community figures to bridge cultural beliefs and modern dental practices. For example, educational programs that frame oral hygiene within the context of spiritual balance and respect for one's *orí* could resonate with yorùbá individuals, encouraging preventive dental practices without dismissing cultural values.

Culturally resonant musical approaches can bridge gaps in dental awareness, making oral health practices more relatable and accessible within these communities. Songs play a vital role in conveying health messages to people, helping them to remember those messages, inspiring them to take action. Songs have been used to enhance public health knowledge and behaviors (66, 67), and their sustained use to improve dental service utilisation in culturally rooted societies holds great potential.

Health policies should, also, consider integrating traditional healers into oral health awareness programs, enabling them to provide basic oral health information or refer individuals to dental clinics when necessary. Integrating traditional and modern dental practices can significantly improve access and utilization of oral health care in culturally rooted communities. By involving respected traditional healers in oral health awareness, policymakers create a model that respects community norms and supports preventive and curative care. Traditional healers' endorsement of practices like fluoride use and timely referrals for complex issues can increase the acceptability of modern dental care, bridging cultural gaps and fostering trust. This collaboration encourages earlier clinic visits and reduces health complications, promoting a holistic, culturally aligned approach to oral health that validates traditional practices while enhancing preventive and professional care.

In addition to provision of care, healthcare professionals (HCPs) have to consider the cultural background while estimating the prevalence of oral diseases. This prevalence is bound to be an underestimation of the true level since people will under report oral diseases and practices that are not socially accepted or that are associated with stigmas. HCPs also have to keep the cultural background in mind while providing oral health instructions and offering advice by acknowledging the cultural background and finding culturally-appropriate solutions to the problems.

Furthermore, acknowledging, respecting, and supporting self-care practices while promoting the complementary benefits of professional dental services can improve individual oral health outcomes. Public health campaigns can help convey messages that promote modern dental care practice as enhancers rather than replacement of these valued practices. In addition, incorporating culturally competent training for HCPs may foster greater understanding of the role self-reliance plays in health behaviors, helping to encourage earlier utilization of preventive and curative services in a way that resonates with cultural values.

Finally, addressing socioeconomic barriers is essential. Subsidizing dental care for underserved communities, improving access to affordable dental care, and ensuring that dental clinics are accessible in rural and urban areas can help reduce financial and logistical barriers. Providing mobile dental services or community-based oral health screenings could also improve access in areas where traditional beliefs are strong and healthcare infrastructure is limited.

## Conclusion

Oral health service utilization in Africa may be influenced by a complex interplay of cultural beliefs, economic constraints, and structural barriers. As the reflection of the yorùbá tradition portrays, though the mouth has spiritual and social significance that highlights the need for its care, the poor uptake of dental care services in the region inhabited by the yorùbá population in Nigeria does not reflect the prime importance the culture seems to place on oral health. This discordance may reflect a preference for traditional oral remedies and, in some cases, reluctance to seek professional dental care based on cultural nuances poorly captured in the narrative about dental service utilisation by the population. By understanding and respecting cultural nuances that may influence the use of orthodox dental care services, healthcare policies and interventions can be better tailored to meet the needs of the populations in Africa where health behaviors are deeply rooted in cultural beliefs and practices. Such an approach may foster a more inclusive approach to oral healthcare that uses traditional practices to support modern dental care.

## Data Availability

The datasets presented in this study can be found in online repositories. The names of the repository/repositories and accession number(s) can be found in the article/Supplementary Material.
